# Can nutritional literacy be an important barrier for obesity and chronic diseases?

**DOI:** 10.1097/MD.0000000000044464

**Published:** 2025-10-03

**Authors:** Asli Gizem Çapar, Eda Nur Çetiner

**Affiliations:** a Nuh Naci Yazgan University, Kayseri, Turkey.

**Keywords:** adult nutrition literacy, anthropometry, chronic diseases, food consumption, obesity

## Abstract

Adequate nutritional literacy, combined with appropriate food choices, can help protect individuals from chronic diseases and enhance both individual and public health. This study aimed to examine the relationship between the nutritional literacy levels of patients receiving dietary counseling at a diet outpatient clinic and their likelihood of being obese or having chronic diseases. The study included 130 patients with chronic diseases (chronic disease group) and 99 healthy individuals (control group), aged 19 to 64. Participants’ nutritional status was assessed using food consumption records. Waist circumference was measured, and body mass index (BMI, kg/m^2^) was calculated. Nutritional literacy levels were evaluated using the Evaluation Instrument of Nutrition Literacy on Adults (EINLA) in all participants. The mean age was 44.0 (32–53) years in the chronic disease group and 42.5 (37–50) years in the control group. Female participants constituted 55.4% (n = 72) of the chronic disease group and 63.6% (n = 63) of the control group. The mean BMI was 27.5 (24.4–30.5) in the chronic disease group and 27.0 (24.3–29.6) in the control group. Chronic diseases among participants included hypertension, diabetes mellitus, heart diseases, chronic obstructive pulmonary disease, asthma, goiter, and obesity. The chronic disease group had significantly higher EINLA scores (26.41 ± 4.94) compared to the control group (24.15 ± 5.62); however, their scores for general nutritional knowledge, reading comprehension, food label reading, and numerical literacy were significantly lower (*P* < .05). EINLA score (1.09 [1.03–1.14], *P* = .002), general nutrition knowledge (odds ratio [OR] = 0.81 [0.68–0.96], *P* = .018), reading comprehension-interpretation (OR = 0.45 [0.32–0.64], *P* < .001), and reading food labels-numerical literacy (OR = 0.77 [0.67–0.90], *P* = .001) were significantly associated with the likelihood of having a nutrition-related chronic disease. These associations remained significant after adjustment. Individuals with adequate nutritional literacy, as measured by the EINLA, exhibited lower BMI. Among those with chronic diseases, higher EINLA scores were inversely associated with obesity risk but positively associated with the presence of nutrition-related chronic diseases, even after adjusting for confounding factors. Furthermore, higher scores in specific EINLA subdimensions were significantly and inversely related to nutrition-related chronic disease presence. These findings underscore the complex relationship between nutritional literacy and health outcomes and suggest the possibility of reverse causality in cross-sectional studies. These findings suggest that higher nutritional literacy, particularly through targeted education on specific subdomains may act as a protective factor against obesity and chronic diseases. Promoting nutritional literacy could be a simple and cost-effective strategy to reduce the public health burden of these conditions.

## 1. Introduction

Several factors, including rapid and unplanned urbanization, unhealthy living conditions, and an aging population, contribute to the increased mortality from chronic diseases.^[1,2]^ Additionally, poor diet and sedentary lifestyles elevate the risk of high blood pressure, blood glucose levels, blood lipids, and noncommunicable diseases (NCDs), such as obesity.^[3]^ In Turkey, similar to developed societies, mortality from NCDs is rising.^[4,5]^ If this trend continues, the growing elderly population, along with increased deaths from chronic diseases, work incapacity, and the demand for social care, will also rise.^[6]^

Today, urbanization, economic development, and globalization have driven lifestyle changes that have altered eating habits. As processed food consumption rises, individuals are consuming foods high in energy, fat, and sodium, while their intake of fruits, vegetables, and fiber declines.^[7]^ Globally, 43% of adults were overweight and 16% were living with obesity in 2022.^[8]^ Similarly to the global trend, the prevalence of obesity in Turkey has also increased, reaching 21.2%.^[4]^ Additionally, the incidence of chronic diseases continues to rise in Turkey.^[9–11]^ It is reported that a healthy diet and the selection of appropriate foods can protect individuals from chronic diseases, reducing health expenditures and economic burden.^[12]^ Therefore, it is crucial for individuals to possess sufficient nutritional knowledge for both personal and public health.^[13]^ Adequate nutritional knowledge may help protect individuals from chronic diseases and promotes both personal and public health by enabling appropriate food choices.^[14]^

Nutritional literacy is defined as the degree to which individuals have the ability to acquire, process, and understand nutritional knowledge and skills.^[15]^ Health literacy is an important factor in preventing diseases related to specific conditions; however, it is not a sufficient indicator for nutrition-related chronic diseases. In light of this, various nutritional literacy scales have been developed in different countries, including the Nutrition Literacy Assessment Instrument for Italian participants (NLit-IT), the Nutrition Literacy Assessment Instrument for Brazilians (NLit-Br), and the Nutrition Literacy Questionnaire (NLQ).^[13,16–20]^

Research conducted on various groups, including children, adolescents, students, and adults, has shown that individuals with higher nutritional literacy tend to have better diet quality and health status compared to those with inadequate nutritional literacy.^[17,21–24]^ Additionally, it has been reported that healthy eating habits^[25]^ and the preference for healthy foods while shopping^[26]^ increase with the rise in nutrition literacy (NL) level. Unhealthy dietary preferences stemming from low nutritional literacy and poor diet quality in individuals increase the prevalence, health burden, cost, and duration of treatment of nutrition-related chronic diseases.^[14,27]^ Socioeconomic inequalities, low levels of education, and household income^[14]^ have been negatively associated with nutritional literacy.^[28,29]^ Furthermore, when examining the relationship between nutritional literacy and anthropometric measurements, it has been found that as nutritional literacy decreases, body fat percentage, body mass index (BMI),^[30]^ waist-hip ratio, and waist-height ratio increase.^[24]^ Hence, the effectiveness of nutritional literacy in promoting healthy eating, protecting, and enhancing health is evident.^[31]^

Nutritional literacy has been extensively studied, especially among healthy individuals and in relation to diseases such as diabetes and cancer.^[14,23,24,32–34]^ For example, Kalkan et al, in a large-scale study conducted in Turkey, found that individuals with low nutritional literacy had significantly higher cardiovascular and diabetes risk scores.^[35]^ Similarly, a study among white-collar workers in Turkey reported that higher nutritional literacy was associated with better diet quality and quality of life.^[36]^ Since most chronic diseases require lifelong nutritional therapy, assessing the nutritional literacy of these patients is critically important. However, to the best of our knowledge, only a limited number of studies have addressed this issue in patients with chronic diseases in Turkey. This study aimed to assess the nutritional literacy levels of individuals with chronic diseases and to examine their association with obesity and the presence of chronic conditions. We hypothesized that low nutritional literacy is associated with a higher likelihood of chronic diseases and obesity.

## 2. Methods

### 2.1. Study design and sample selection

This study was designed as a cross-sectional analysis aimed at assessing the nutritional literacy levels of individuals with nutrition-related chronic diseases and examining their associations with dietary and anthropometric characteristics. Although a control group was included for comparison, the study was not designed as a case-control study. No matching or stratification procedures were applied during participant recruitment. The control group consisted of individuals without any diagnosed nutrition-related chronic diseases but with similar sociodemographic characteristics (e.g., age, gender, education level) to the chronic disease group. The purpose of including this group was to evaluate and compare nutritional literacy levels in individuals with and without chronic disease, rather than to perform matched pair analyses. Therefore, all data were analyzed within the framework of a cross-sectional design.

This cross-sectional study was conducted between March 2022 and June 2022 with patients who visited the Diet Polyclinic at Erciyes University Medical Faculty Training and Research Hospital due to chronic diseases (hypertension, diabetes mellitus, heart diseases, chronic obstructive pulmonary disease, asthma, goiter, obesity), as well as healthy individuals. The sample size in our study was calculated based on a prior study by Kamarli Altun et al,^[37]^ which compared NL scores between male and female adults. In that study, the mean NL score was reported as 27.9 ± 2.76 for females and 26.5 ± 3.32 for males, with a statistically significant difference (*P* < .001).

Using these figures, an effect size (Cohen *d*) of approximately 0.439 was estimated. A power analysis was conducted with α = 0.01 and power = 0.95 (β = 0.05), indicating a required minimum sample size of 96 participants for a 2-tailed independent samples *t*-test.

Given the similarity of our target population and study design, this calculation was used as the basis for estimating the sample size in our own study.

This study included 130 adults who visited the outpatient clinic for nutritional therapy due to chronic diseases and 99 healthy controls. The exclusion criteria were being <19 or >64 years of age, pregnancy, and the inability to answer the survey questions.

Ethics committee approval (decision number 2022/6546, dated 2 August 2022) and institutional permission were obtained from the Nuh Naci Yazgan University Ethics Committee. Participants were provided with an informed consent form, which they were asked to read and sign voluntarily before completing the survey. This study was conducted in accordance with the ethical standards outlined in the Declaration of Helsinki.

### 2.2. Data collection process

The data for the study were collected using a face-to-face data collection method with an 11-question questionnaire. The questionnaire included items on sociodemographic characteristics, chronic diseases, reasons for referral to the diet polyclinic, prescribed diet therapy, and previous diet history, in accordance with the literature. The survey took approximately 8 to 10 minutes to complete.

**Figure 1.**
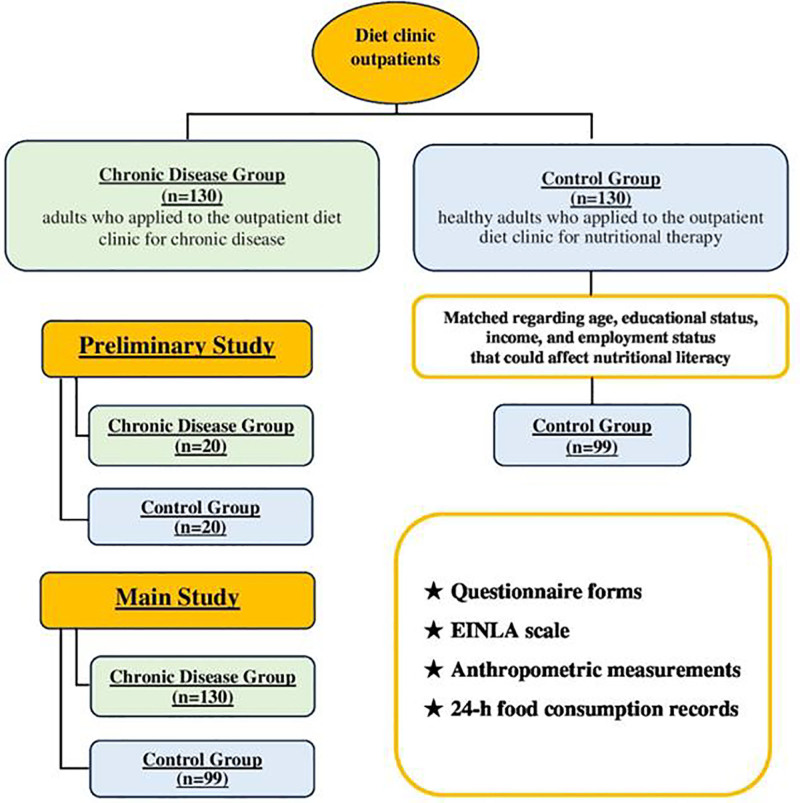
Participant flowchart. EINLA = Evaluation Instrument of Nutrition Literacy on Adults.

The functionality of the pre-study questionnaire was tested on 20 chronic patients and 20 control participants, who were not included in the study. Additionally, the data obtained from the pilot study were not included in the main analyses but were used to evaluate the internal consistency of the questionnaire. The Cronbach alpha coefficient was found to be 0.767. The final study sample consisted of 260 individuals, including 130 patients (chronic disease group) seeking nutritional therapy for their chronic conditions and 130 healthy controls selected from hospital staff by the researcher. After matching for age, educational status, income, and employment status (factors that could affect nutritional literacy), the control group was reduced to 99 participants. The participant flow and the selection process for the study groups are shown in Figure 1.

### 2.3. Anthropometric measurements

The body height, body weight, and waist circumference (WC) of all participants were measured by the researchers at the diet polyclinic following standard measurement protocols. Height was measured using a stadiometer, with participants standing without shoes, heels together, and feet positioned at a 45° angle, and the head aligned with the Frankfort plane (SECA Ltd., Hamburg, Germany). Body weight was measured using a precise standard weighing scale with an accuracy of 0.1 kg (SECA Ltd., Hamburg, Germany). BMI was calculated using the formula: BMI = weight (kg)/height^2^ (m^2^). Based on the WHO adult BMI classification, participants were categorized as underweight (BMI < 18.5 kg/m^2^), normal weight (BMI 18.5–24.9 kg/m^2^), overweight (BMI 25–29.9 kg/m^2^), and obese (BMI ≥ 30 kg/m^2^).^[38]^ WC was measured using a nonelastic tape measure at the level of the lateral iliac crest and the midpoint of the lowest rib, while participants stood without clothing. Each measurement was taken twice, and if the 2 values differed by no more than 1 cm, their average was used to ensure consistency. If the difference exceeded 1 cm, the measurements were repeated.^[39]^

### 2.4. Evaluation Instrument of Nutrition Literacy on Adults (EINLA)

Many scales have been developed to measure health literacy, specific to diseases and in healthy individuals. However, since a validity and reliability study has been conducted in Turkish, EINLA was used in this research. EINLA was developed by Gibbs and Chapman-Novakofski.^[40]^ The Turkish version of EINLA was adapted by Cesur et al.^[16]^ It consists of 5 subsections and 35 questions, including “general nutritional knowledge,” “reading comprehension and interpretation,” “food groups,” “portion amounts,” and “reading food labels-numerical literacy.” This version was used to determine the nutritional literacy levels of the participants. Permission was obtained from the authors to use this scale. Each correct answer received 1 point, while incorrect or unanswered questions received 0 points. The total score was categorized as follows: 0 to 11 points indicated an inadequate level, 12 to 23 points indicated a limited level, and 24 to 35 points indicated an adequate level of nutritional literacy. The score levels and classifications for each subsection varied. In the first section, which covered general nutritional knowledge, 0 to 3 points indicated an insufficient level, 4 to 7 points indicated a limited level, and 8 to 10 points indicated an adequate level. The second section, focused on reading comprehension and interpretation, had 6 questions. Here, 0 to 2 points indicated insufficient literacy, 3 to 4 points indicated a limited level, and 5 to 6 points indicated sufficient literacy. The third section covered food groups and had 10 questions. In this section, 0 to 3 points indicated an insufficient level, 4 to 7 points indicated a limited level, and 8 to 10 points indicated sufficient literacy. The fourth section contained 3 questions about portion amounts, where 0 to 1 points indicated insufficient literacy, 2 points indicated a limited level, and 3 points indicated sufficient literacy. Finally, the fifth section, about reading food labels and numerical literacy, consisted of 6 questions. For this section, 0 to 2 points indicated insufficient literacy, 3 to 4 points indicated a limited level, and 5 to 6 points indicated sufficient literacy. The total response time for the entire questionnaire was approximately 20 minutes.

### 2.5. Determination of daily energy and macro nutrient intakes

In food consumption studies, a 24-hour recall is widely used to obtain accurate and representative data.^[41]^ Accordingly, this method was applied in this study to estimate the participants’ energy and nutrient intakes, consistent with similar studies reviewed in the literature.^[35,42–44]^ Due to the design of our study, dietary intake data were collected only during the outpatient clinic visit, which limited the records to a single day. To accurately determine the portion amounts of the meals consumed by individuals, the “Food and Nutrition Photo Catalogue” was utilized.^[45]^ The daily energy and macronutrient intakes of individuals, based on the 24-hour recall food consumption record, were calculated using the Nutrition Information Systems Package Program (BEBIS 8.1 Full version), developed at Hohenheim University, Stuttgart, Germany.^[46]^

### 2.6. Statistical analyses

The data were analyzed using SPSS 22.0 (Statistical Package for Social Sciences Statistics, SPSS IBM Corp., Chicago). Descriptive findings were expressed as numbers and percentages. The normality of the data distribution was assessed using histograms, Q-Q plots, and the Shapiro–Wilk test. The relationship between categorical variables was assessed using the Pearson chi-square test. The comparison between independent groups was evaluated using the independent *t*-test or the Mann–Whitney *U* test, depending on whether the data followed a normal distribution. The Kruskal–Wallis test was used for comparisons among more than 2 groups. For multiple comparisons, the Bonferroni correction was applied. Spearman correlation analysis was used to examine the relationship between nutritional literacy and daily energy and macronutrient intakes. Multiple logistic regression was applied to assess the relationship between nutritional literacy scores and obesity, based on BMI, while binary logistic regression analysis was conducted to determine the likelihood of developing a nutrition-related chronic disease (n = 130). For these analyses, 2 models were established: the raw model of linear regression did not account for confounding variables whereas the adjusted model included age, gender, education level, and income as covariates, in line with findings from previous studies.^[47–50]^ In the binary logistic regression analysis, BMI was included as a confounding factor. Additionally, based on preliminary analyses of the dataset, an alternative linear regression model was developed in which only gender and income status were included as covariates, reflecting their observed influence on the outcome variables. Following the literature review, we included age, gender, education, and income as covariates in one of the models. For the alternative model, gender and income were included as covariates based on both preliminary data exploration and evidence from prior studies suggesting their potential confounding effects. Odds ratios were reported with 95% confidence intervals. A correlation coefficient absolute value between *R* = 0.00 and 0.50 was considered weak, and between *R* = 0.50 and 1.0 was considered strong.^[51]^ Additionally, *P*-values <.2 in the regression analyses were considered indicative of a potential relationship, even if they were not statistically significant.^[52]^ A *P*-value <.05 was considered statistically significant.

## 3. Results

### 3.1. Demographic and basic characteristics of participants

The median age of the participants was 43 (35–53) years, and 58.95% (n = 135) of the participants were women. The age, gender, BMI, education, employment and income status, as well as the levels of daily energy and macronutrient intake, were similar across both groups. The percentage of participants with limited nutritional literacy was 52.5% in the control group and 28.5% in the other group (*P* < .001, Table 1). A total of 35.4% of patients presented at the diet clinic with a diagnosis of diabetes mellitus, 13.8% with multiple chronic diseases, and 13.8% with hypertension (Table 1).

**Table 1:** Comparison of socio-demographical characteristics of the groups and energy and macro nutrient intake and some anthropometric measurement values (n = 229).

	Chronic disease group (n = 130)	Control group (n = 99)	*P*
Gender, n (%)*			
Male	58 (44.6)	36 (36.4)	.209
Female	72 (55.4)	63 (63.6)	
Chronic disease, n (%)			
Hypertension	18(13.8)	–	–
Diabetes mellitus	46 (35.4)		
Heart diseases	16 (12.4)		
COPD	11 (8.5)		
Asthma	6 (4.6)		
Goiter	6 (4.6)		
Obesity	9 (6.9)		
Multiple chronic diseases	18 (13.8)		
Age (yr) (median (IQR))†	44.0 (32–53)	42.5 (37–50)	.718
Weight (kg) ($\bar{x}\pm SD$)	78.05 ± 15.06	76.66 ± 14.36	.480
BMI (kg/m^2^) (median (IQR))†	27.5 (24.4–30.5)	27.0 (24.3–29.6)	.363
WC (cm) (median (IQR))†	93.5 (80.7–106.0)	90.0 (80.0–100.0)	.110
Energy (kcal) (median (IQR))	1569 (483–3669)	1582 (578–3859)	.754
Carbohydrate (g) (median (IQR))	185.3 (17.5–596.9)	167.4 (41.3–451.4)	.085
Protein (g) (median (IQR))	64.7 (13.0–173.8)	66.48 (16.8–148.9)	.289
Lipid (g) (median (IQR))	64.6 (18.9–184.8)	69.98 (17.2–191.6)	.235
BMI classification, n (%)*			
Underweight	2 (1.5)	1 (1.0)	.748
Normal	33 (25.4)	27 (27.3)	
Overweight	57 (43.9)	48 (48.5)	
Obese	38 (29.2)	23 (23.2)	
Education status, n (%)*			
Primary education	47 (36.2)	34 (34.3)	.915
High school	40 (30.8)	33 (33.3)	
University graduation	43 (33.0)	32 (32.4)	
Working status, n (%)*			
Yes	61 (46.9)	38 (38.4)	.244
No	69 (53.1)	61 (61.6)	
Income status, n (%)*			
Income more than expenses	31 (23.9)	19 (19.2)	.667
Income equals expense	69 (53.0)	59 (59.6)	
Income less than expense	30 (23.1)	21 (21.2)	
EINLA, n (%)*			
Limited level	37 (28.5)	52 (52.5)	**<.001**
Adequate level	93 (71.5)	47 (47.5)	

BMI = body mass index, COPD = chronic obstructive pulmonary disease, EINLA = Evaluation Instrument of Nutrition Literacy on Adults, IQR = interquartile range, WC = waist circumference. Values in bold indicate *P* < .05, which is considered a significant difference.

* Chi-square test and Student *t* test.

† Mann–Whitney *U* test.

### 3.2. Physical and socioeconomic variables according to nutritional literacy

The age (*P* = .007), body weight (*P* < .001), BMI (*P* = .012), and WC (*P* = .003) of individuals with limited nutritional literacy were higher than those with adequate nutritional literacy, but income levels were lower (*P* = .046). However, there were no significant differences between individuals with limited and adequate nutritional literacy in terms of energy and macronutrient intake, BMI classification, education, and employment status.

### 3.3. Comparison of NL and subscale scores across different groups based on gender, education, and income level

Although the total EINLA score of the chronic disease patient group was higher than that of the control group, scores for general nutritional knowledge, reading comprehension and interpretation, reading food labels, and numerical literacy were significantly lower (*P* < .05). The total EINLA score was significantly higher among women, university graduates, and individuals with higher income levels compared to other groups (*P* < .05). In the subscale analyses, women and university graduates had higher scores in the general nutrition knowledge subscale; university graduates scored higher than primary school graduates in the reading comprehension and interpretation subscale; and university graduates and individuals with higher income levels had significantly higher scores in the reading food labels-numerical literacy subscale compared to other groups (*P* < .05).

### 3.4. Associations between nutrient intake and nutritional knowledge

In the chronic disease group, statistically significant and weak negative correlations were observed between the EINLA score and carbohydrate intake (*r* = −0.180, *P* < .05). In both the chronic disease and control groups, general nutrition knowledge scores were weakly and negatively correlated with carbohydrate intake, with the correlation reaching statistical significance (*P* < .05). In the control group, a statistically significant and weak negative correlation was observed between general nutrition knowledge and protein intake (*r* = −0.220, *P* < .05) (Table 2).

**Table 2:** The relationship between EINLA and subdimensions scores and daily energy and macronutrient intake of both groups (n = 229).

Variables	EINLA		General nutrition knowledge		Reading comprehension and interpretation		Food groups		Portion amounts		Reading food labels-numerical literacy	
	CD	CG	CD	CG	CD	CG	CD	CG	CD	CG	CD	CG
Energy (kcal)	−0.06	0.10	−0.15	−0.20	−0.08	−0.003	−0.10	0.13	−0.02	0.06	0.01	0.18
CHO (g)	−0.18*	0.16	−0.26**	−0.23*	−0.11	−0.04	−0.15	0.095	−0.06	0.06	−0.08	0.15
Protein (g)	0.003	−0.06	−0.11	−0.22*	−0.02	0.01	−0.03	0.02	−0.01	0.16	0.10	0.07
Lipid (g)	0.13	0.05	0.06	−0.05	−0.03	0.31	0.05	0.17	−0.01	0.01	0.14	0.20

Data in the table show Spearman correlation coefficients (rho).

CD = chronic disease group, CG = control group, CHO = Carbohydrate(s), EINLA = Evaluation Instrument of Nutrition Literacy on Adults.

* *P* < .05, Spearman correlation analysis.

** *P* < .01, Spearman correlation analysis.

### 3.5. Relationship between EINLA scores and BMI in the chronic disease group

According to the linear regression analysis evaluating the relationship between BMI and EINLA and its subdimensions, a negative and statistically significant association was observed between total EINLA score and the probability of obesity among individuals with chronic diseases (n = 130). This association remained significant in the unadjusted (raw) model (β = −0.203, *P* = .042) and in Model 1, which was adjusted for gender and income (β = −0.161, *P* = .013). However, the association lost its significance in Model 2, which additionally controlled for age and educational status (β = −0.154, *P* = .151). Among the subdimensions, portion size knowledge was significantly and inversely associated with obesity in Model 1 (β = −0.933, *P* = .028). Similarly, food label reading and numerical literacy scores showed a significant negative association with the probability of obesity in both the raw model (β = −0.544, *P* = .027) and Model 1 (β = −0.445, *P* = .014). However, these associations were no longer significant in Model 2 (*P* > .05). Other subdimensions (including general nutrition knowledge, reading comprehension and interpretation, and knowledge of food groups) were not significantly associated with obesity in any of the models (*P* > .05) (Table 3).

**Table 3:** The relationship with EINLA and its subdimensions scores and the probability of obese of participants according to BMI of chronic disease group (n = 130).

	BMI (kg/m^2^)				
	Coefficient (beta)	95% confidence interval		*R* ^2^	*P*
		Lower	Upper		
EINLA					
Raw model	−0.203	−0.397	−0.008	0.179	**.042**
Model 1	−0.161	−0.288	−0.035	0.178	**.013**
Model 2	−0.154	−−0.365	0.057	0.320	.151
General nutrition knowledge					
Raw model	−0.214	−0.804	0.377	0.063	.475
Model 1	−0.201	−0.641	0.240	0.088	.370
Model 2	−0.122	−0.734	0.490	0.297	.694
Reading comprehension and interpretation					
Raw model	−0.240	−1.109	0.629	0.048	.586
Model 1	−0.482	−1.124	0.161	0.117	.141
Model 2	−0.228	−0.896	0.441	0.258	.503
Food groups					
Raw model	−0.402	−0.956	0.151	0.126	.153
Model 1	−0.158	−0.564	0.248	0.082	.443
Model 2	−0.390	−0.942	0.162	0.318	.165
Portion amounts					
Raw model	−0.874	−2.039	0.291	0.130	.140
Model 1	−0.933	−1.765	−0.101	0.160	**.028**
Model 2	−0.670	−1.875	0.534	0.310	.273
Reading food labels-numerical literacy					
Raw model	−0.544	−1.024	−0.064	0.195	**.027**
Model 1	−0.445	−0.801	−0.090	0.175	**.014**
Model 2	−0.382	−0.889	0.125	0.321	.139

Linear regression analysis. Model 1: adjustments were made for gender and income. Model 2: Adjustments were made for age, gender, income, and education status.

BMI = body mass index, EINLA = Evaluation Instrument of Nutrition Literacy on Adults. Values in bold indicate *P* < .05, which is considered a significant difference.

### 3.6. Association of nutrition-related chronic diseases with EINLA and subdimensions in the chronic disease group

Binary logistic regression analysis was performed to evaluate the association between total EINLA scores and its subdimensions and the presence of nutrition-related chronic diseases among individuals in the chronic disease group (n = 130). The results indicated that higher total EINLA scores were significantly and positively associated with the presence of a nutrition-related chronic disease in both the unadjusted model (odds ratio [OR] = 1.09, *P* = .002) and in Model 1, which was adjusted for age, gender, income, educational status, and BMI (OR = 1.09, *P* = .002). Conversely, higher scores in certain EINLA subdimensions were significantly and inversely associated with the presence of nutrition-related chronic disease. General nutrition knowledge was associated with lower odds of having a chronic disease in both the raw model (OR = 0.81, *P* = .018) and Model 1 (OR = 0.80, *P* = .031). Reading comprehension and interpretation showed the strongest inverse association (raw model: OR = 0.45, *P* < .001; Model 1: OR = 0.40, *P* < .001). Similarly, food label reading and numerical literacy was significantly associated with reduced odds of disease presence in both models (raw model: OR = 0.77, *P* = .001; Model 1: OR = 0.74, *P* = .001) (Table 4).

**Table 4:** The relationship with EINLA and its subdimensions scores and the probability of presence of nutrition-related chronic disease of chronic disease group (n = 130).

	The presence of nutrition-related chronic disease	
	OR in the EINLA and subdimensions (95% confidence interval)	*P*
EINLA		
Raw model	1.09 (1.03–1.14)	**.002**
Model 1	1.09 (1.03–1.16)	**.002**
General nutrition knowledge		
Raw model	0.81 (0.68–0.96)	**.018**
Model 1	0.80 (0.66–0.98)	**.031**
Reading comprehension and interpretation		
Raw model	0.45 (0.32–0.64)	**<.001**
Model 1	0.40 (0.27–0.60)	**<.001**
Food groups		
Raw model	0.89 (0.75–1.06)	.183
Model 1	0.91 (0.76–1.08)	.280
Portion amounts		
Raw model	1.06 (0.76–1.46)	.746
Model 1	1.07 (0.76–1.51)	.703
Reading food labels-numerical literacy		
Raw model	0.77 (0.67–0.90)	**.001**
Model 1	0.74 (0.63–0.88)	**.001**

Binary logistic regression analysis. Model 1: Adjustments were made for gender, age, income and educational status, and BMI.

BMI = body mass index, EINLA = Evaluation Instrument of Nutrition Literacy on Adults, OR = odds ratio. Values in bold indicate *P* < .05, which is considered a significant difference.

## 4. Discussion

Considering the importance of lifelong nutritional therapy in the management of chronic diseases, assessing the nutritional literacy levels of these patients emerges as a critical necessity. Although previous studies have associated nutritional literacy with nutritional status and healthy eating habits,^[53]^ this issue has not yet been sufficiently investigated in chronic patients.^[14,28]^ This study aimed to assess the nutrition literacy levels of individuals with chronic diseases and to examine their association with obesity and the presence of chronic conditions. Additionally, the relationships between nutritional literacy, selected sociodemographic factors, and nutritional status were evaluated. The main findings of this study indicate that total EINLA scores are significantly and positively associated with the presence of nutrition-related chronic diseases, even after adjusting for age, gender, income, educational status, and BMI. Conversely, higher scores in certain EINLA subdimensions show significant inverse associations with the presence of nutrition-related chronic diseases. These results suggest a complex relationship between overall NL and specific components of nutrition-related knowledge in relation to chronic disease status. Furthermore, an inverse relationship was observed between NL levels and obesity risk, indicating that higher NL may be associated with a lower likelihood of obesity. While daily nutrient intakes were similar across groups categorized by NL levels, a negative association was observed between carbohydrate intake and both total EINLA score and general nutrition knowledge within the chronic disease group; that is, higher general nutrition knowledge was associated with lower carbohydrate consumption.

Currently, it is suggested that, due to increased access to the internet among young people, there might be a higher level of nutritional knowledge as access to information becomes more accessible.^[22]^ Studies have indicated that individuals with lower nutritional literacy tend to be older, have lower educational levels, lower household incomes, and are more susceptible to receiving a diagnosis of chronic disease (type 2 diabetes).^[14,54,55]^ In this study, among the chronic disease group, 35.4% of the patients had type 2 diabetes, 12.4% had cardiovascular disease, 13.8% had hypertension, and 13.8% had multiple chronic disease (Table 1). Even though the age, gender, education, and income status were similar between the groups, it was observed that the age of individuals at a limited level was higher than those at an adequate level, but income levels were lower. Additionally, the total EINLA score was significantly higher among women, university graduates, and individuals with higher income levels compared to other groups (*P* < .05) (Table 5). These findings were consistent with previous study results.^[25,28,29,54]^

**Table 5:** Comparison of nutrition literacy and subscale scores across different groups based on gender, education, and income level (n = 229).

	EINLA	General nutrition knowledge	Reading comprehension and interpretation	Food groups	Portion amounts	Reading food labels-numerical literacy
Groups						
Chronic disease group (n = 130)	26.41 ± 4.94	7.85 ± 1.65	4.74 ± 1.12	8.88 ± 1.75	1.79 ± 0.83	3.36 ± 2.00
Control group (n = 99)	24.15 ± 5.62	8.34 ± 1.38	5.38 ± 0.77	9.17 ± 1.48	1.76 ± 0.77	4.21 ± 1.57
Total group	25.43 ± 5.35	8.06 ± 1.56	5.02 ± 1.03	9.00 ± 1.64	1.77 ± 0.80	3.72 ± 1.87
*P, t*	**.001**, 3.225	**.016,** 2.419	**<.001**, 4.909	.179, 1.348	.747, 0.323	**<.001**, 3.488
Gender						
Female (n = 72)	27.5 (23.3–31.0)	9.0 (7.0–9.0)	5.0 (4.0–5.0)	10.0 (9.0–10.0)	2.0 (1.0–3.0)	3.5 (2.0–6.0)
Male (n = 58)	27.0 (22.0–29.0)	7.5 (6.0–9.0)	5.0 (4.0–5.3)	10.0 (8.0–10.0)	2.0 (1.0–2.0)	3.5 (2.0–5.0)
*P, U*	**.033**, 1633.5	**.010**, 1551.0	.668, 2002.0	.424, 1931.5	**.006**, 1538.5	.517, 1951.5
Education status						
Primary education (n = 47)	24.0 (21.0–27.0)^a^	7.0 (6.0–8.0)^a^	5.0 (4.0–5.0)^a^	9.0 (8.0–10.0)	2.0 (1.0–2.0)	2.0 (1.0–4.0)^a^
High school (n = 40)	27.0 (23.0–29.8)^b^	8.0 (7.0–9.0)^a^	5.0 (4.0–5.0)^ab^	9.5 (9.0–10.0)	2.0 (1.0–2.8)	4.0 (2.0–5.8)^ab^
University graduation (n = 43)	30.0 (27.0–32.0)^c^	9.0 (8.0–10.0)^b^	5.0 (5.0–6.0)^b^	10.0 (9.0–10.0)	2.0 (2.0–2.0)	5.0 (3.0–6.0)^b^
*P, F*	**<.001**, 26.893	**<.001**, 19.406	**<.001**, 20.358	.319, 2.286	.106, 4. 486	**<.001**, 15.399
Marital status						
Yes (n = 94)	27.0 (23.0–29.3)	8.0 (7.0–9.0)	5.0 (4.0–5.0)	9.5 (8.0–10.0)	2.0 (1.0–2.0)	3.0 (2.0–5.0)
No (n = 36)	28.0 (23.0–32.8)	8.0 (6.3–9.0)	5.0 (4.0–6.0)	10.0 (9.0–10.0)	2.0 (2.0–3.0)	4.0 (2.0–6.0)
*P, U*	.172, 1953.5	.760, 1634.5	.162, 1944.0	.276, 1884.0	**.017**, 2123.0	.258, 1906.5
Working status						
Yes (n = 61)	28.0 (23.0–31.0)	8.0 (7.0–9.0)	5.0 (4.0–6.0)	10.0 (9.0–10.0)	2.0 (1.0–2.0)	4.0 (2.0–5.0)
No (n = 69)	26.0 (23.0–29.0)	8.0 (7.0–9.0)	5.0 (4.0–5.0)	9.0 (8.0–10.0)	2.0 (1.0–2.5)	3.0 (1.0–5.0)
*P, U*	.158, 1802.5	.953, 2117.0	.523, 1976.0	.232, 1870.0	.486, 2245.0	.071, 1722.5
Income status						
Income more than expenses (n = 49)	30.0 (27.0–33.0)^a^	9.0 (8.0–9.0)	5.0 (5.0–6.0)	10.0 (9.0–10.0)^a^	2.0 (1.0–2.0)	5.0 (3.0–6.0)^a^
Income equals expense (n = 126)	26.0 (22.5–29.0)^b^	8.0 (7.0–9.0)	5.0 (4.0–5.0)	9.0 (8.0–10.0)^b^	2.0 (1.0–2.0)	3.0 (1.5–5.0)^b^
Income less than expense (n = 51)	27.0 (22.8–29.0)^b^	8.0 (7.0–9.0)	5.0 (4.0–5.0)	9.0 (8.0–10.0)^ab^	2.0 (1.0–3.0)	2.5 (2.0–4.3)^b^
*P, F*	**.003,** 11.489	.136, 3.994	.064, 5.506	**.046**, 6.167	.470, 1.512	**. 0003**, 11.488

Values are expressed as median (1st–3rd quartiles). Mann–Whitney *U* test, Kruskal–Wallis test, and Student *t* test. The same superscript letters on the same line represent similarity between groups, and different letters represent difference. Values in bold indicate *P* < .05, which is considered a significant difference.

NL levels are positively associated with healthier eating behaviors and greater adherence to the Mediterranean dietary pattern, while lower levels of NL are linked to increased adherence to Western dietary patterns.^[14]^

NL is also associated with chronic diseases such as cardiovascular disease and diabetes, which can be managed through nutritional status.^[35]^ The total EINLA score was positively associated with the presence of nutrition-related chronic disease in both unadjusted and adjusted models controlling for age, gender, income, education, and BMI (Table 4). The observed association between higher total NL scores and the presence of chronic disease is likely attributable to the fact that participants in the chronic disease group had previously been diagnosed and referred to diet outpatient clinics for nutritional therapy. This situation can be interpreted as a reflection of increased awareness and efforts to improve nutrition knowledge among individuals with chronic diseases. Therefore, the elevated NL observed in this group should be considered a consequence of their health status and exposure to nutritional guidance, rather than a cause of the disease. The presence of a positive relationship between overall NL and chronic disease risk is an uncommon yet noteworthy finding. This may suggest that individuals diagnosed with a chronic disease tend to acquire more nutrition-related knowledge following their diagnosis, indicating the possibility of reverse causality.

Obesity being the cornerstone of metabolic diseases,^[56]^ is defined as a chronic disease by the American Association of Clinical Endocrinology and the American Medical Association.^[57,58]^ In Turkey, according to 2019 data, when compared to 2008, the prevalence of obesity increased from 12.3% (2008) to 17.3% in men and from 17.3% (2008) to 18.5% in women.^[10]^ In our study, 26.6% of the participants were obese (this finding was not presented in the table). Although some studies have determined a relationship between the level of nutritional literacy and obesity and BMI,^[58,59]^ this relationship has not been confirmed in other cross-sectional studies conducted in recent years.^[27,60]^ A study conducted in Palestine examined the relationship between nutritional literacy and dietary behaviors among 101 participants with an average age of 22.7 ± 8.7 years.^[25]^ The study utilized the Short Form Diet Health Knowledge Survey for nutritional literacy assessment. The study found a prevalence of adequate nutritional literacy at 29% and determined that 5.7% of the participants were obese. However, the study found no relationship between nutritional literacy and BMI.

According to the EINLA scores obtained in our study, 53.4% of participants with limited nutritional literacy were overweight, and 27.1% were obese, which is higher than the rates reported in the study conducted by Natour et al.^[25]^ It is believed that the higher ages of the participants in our study might have influenced these results. In their 2019 study, Khadem Al-hosseini et al^[61]^ did not find a significant relationship between the level of nutritional literacy and body weight or BMI among 176 participants aged 18 and above. On the other hand, Li et al^[59]^ in their 2022 study in China, aimed to assess the relationship between nutritional literacy and overweight/obesity among 18,176 adolescents. They found that individuals with low nutritional literacy scores (below average) had a higher likelihood of being overweight or obese compared to those with higher scores. Similarly, in this study, in the raw model, it was found that the total EINLA score and BMI (β = −0.203, *P* = .042) were negatively associated. The association remained significant in Model 1, after adjusting for gender and income (β = −0.161, *P* = .013). Among the subdimensions, portion size knowledge was significantly and inversely associated with obesity in Model 1 (β = 0.160, *P* = .028), while food label reading and numerical literacy were significantly and inversely associated with obesity in both the raw model (β = 0.195, *P* = .027) and Model 1 (β = 0.175, *P* = .014). (Table 3). These findings suggest that specific components of NL, particularly portion control and food label literacy, may be associated with obesity among individuals with chronic conditions. However, this relationship appears to be influenced by demographic factors such as age and educational level. Although this relationship was not encountered in the adjusted model, it was assumed that there could still be a significant association with increases in BMI due to the *P*-value being <.2.^[52]^

In different cross-sectional studies conducted on various groups, it is stated that with the increase in the level of nutritional literacy, healthy foods are preferred, and adequate and balanced nutrition is provided, which is effective in the protection and development of health.^[22,31,61]^

In this study, in the chronic disease group, a weak but statistically significant negative correlation was observed between total EINLA scores and carbohydrate intake (*r* = −0.180, *P* < .05). Similarly, in both the chronic disease and control groups, general nutrition knowledge scores were negatively associated with carbohydrate intake, with the correlations reaching statistical significance (*P* < .05) (Table 2). These findings reveal that higher general nutrition knowledge is associated with lower carbohydrate intake in both groups, even though the correlation is relatively weak. This inverse relationship may reflect the widespread belief within the general population that excessive carbohydrate consumption is detrimental to health. Such perceptions, shaped by public health messages and popular dietary trends, could influence individuals with higher nutrition knowledge to intentionally reduce their carbohydrate intake, regardless of their actual health status.^[11,62–64]^

NL includes the skills to understand nutritional information and food groups, read food labels, determine the appropriate amount of food, and ultimately make conscious decisions.^[65]^ As previously mentioned, the EINLA assesses NL through 5 subdimensions, each of which plays a crucial role in shaping individuals’ dietary behaviors and, consequently, in determining their risk for chronic diseases. Gibbs et al reported that individuals with an accurate understanding of food groups tend to have better diet quality, which may help reduce the risk of nutrition-related chronic diseases such as type 2 diabetes and cardiovascular diseases.^[17]^ General nutrition knowledge facilitates the adoption of healthy dietary habits and thus contributes to lowering chronic disease risk. Supporting this, a study conducted by Wang et al in China found an inverse association between higher levels of nutrition and health knowledge and the presence of multiple chronic diseases.^[66]^ Portion control is also a critical factor, particularly in relation to weight management and metabolic syndrome. In a study conducted by Kalkan et al, individuals classified as low-risk for certain chronic diseases were found to have significantly higher scores in the portion control subdimension of the NL scale compared to those in the high-risk group.^[35]^ In this study, a relationship has been demonstrated between reading food labels and a decrease in the risk of nutrition-related NCDs. Enhancing diets through food labeling might directly influence the occurrence and mortality rates of chronic diseases related to nutrition, given the association between dietary intake and chronic disease risk.^[67]^ For instance, increased consumption of fruits and vegetables has been linked to a reduced risk of coronary heart disease.^[68]^ Additionally, higher fiber intake has been connected to a decreased risk of both colorectal cancer and stroke incidence.^[69,70]^ Conversely, elevated salt intake has shown a positive correlation with blood pressure, a factor closely tied to the risk of stroke and coronary heart disease.^[71]^ In a cross-sectional study evaluating the relationship between food labels and chronic disease, it was found that awareness or use of food labels is associated with lower cardiovascular disease (coronary heart disease, stroke, heart failure, hypertensive disease, and lung and colorectal cancers) mortality and delayed occurrence of deaths.^[72]^ In this study, the analysis of EINLA subdimensions, it was observed that specifically reading comprehension and interpretation skills (OR: 0.40, *P* < .001) as well as food label reading and numerical literacy (OR: 0.74, *P* = .001) were significantly associated with a protective effect against the risk of nutrition-related chronic diseases (Table 4). In a study examining the relationship between nutrition labels and chronic diseases, disease awareness was found to be associated with the use of nutrition labels. The importance of individuals’ use of nutrition labels in the management of chronic diseases was emphasized.^[73]^ Indeed, previous literature has highlighted that health behavior changes often occur after disease onset, and the potential for reverse causality should be considered in such associations.^[66]^

Nevertheless, specific subdomains, such as reading comprehension and food label literacy, may have a protective role in the prevention or management of these conditions (Table 4). Therefore, we believe that having sufficient nutritional literacy can contribute to preventing obesity and nutrition-related chronic diseases and reducing the economic, social, and health burdens they bring. It is, therefore, crucial that the issue of NL be evaluated and developed by policymakers, researchers, and other stakeholders in society. Integrating NL enhancement into public health policies and clinical practices is essential to effectively reduce the burden of diet-related chronic diseases. Additionally, dietitians should actively raise awareness about the importance of NL and play a key role in educating the public.

### 4.1. Limitations

This study presents several limitations that should be acknowledged. First, the relatively small sample size may limit the generalizability of the findings. Second, since the study population consists of adults residing in Turkey, the results may not be generalizable to populations with different cultural or geographical backgrounds. Furthermore, although the 24-hour dietary recall method is widely used in nutritional research, it reflects only a short-term dietary intake and may not accurately capture participants’ usual eating behaviors. Therefore, future studies employing longitudinal designs with larger and more diverse populations are recommended to provide a clearer understanding of the associations between nutritional literacy, obesity, and chronic disease risk.

## 5. Conclusions

The first important finding is that total EINLA scores showed a significant positive association with the presence of nutrition-related chronic diseases even after adjusting for age, gender, income, educational status, and BMI. This suggests a complex relationship between overall NL and chronic disease status. Furthermore, it indicates that NL may have increased following diagnosis among affected individuals. The second key point was the potential decrease in BMI with an increase in nutritional literacy scores. Thirdly, although individuals with chronic diseases tended to have lower scores in the subdimensions of general nutrition knowledge, reading comprehension and interpretation, and food label reading-numerical literacy, higher scores in these specific EINLA subdimensions were significantly and inversely associated with the presence of nutrition-related chronic diseases. This finding suggests that proficiency in these particular domains of nutritional literacy may serve a protective role and highlights their potential importance in the prevention and management of chronic diseases. However, further research with larger sample sizes on this matter is considered valuable for public health. These findings could influence the development or modification of public health policies related to nutrition education, access to healthier foods, or interventions aimed at reducing the incidence of chronic diseases. Health professionals could use these findings to emphasize the importance of nutritional education and literacy in managing and preventing chronic diseases. Additionally, schools, community centers, or healthcare facilities could implement programs to improve nutritional literacy among various age groups.

## Acknowledgments

The authors would like to thank all participants who devoted their time to participate in this study and the students of 4th who collected data. The authors would like to express their gratitude to Neriman İnanç from the Department of Nutrition and Dietetics in Nuh Naci Yazgan University for their insightful comments and consultancy. We would also like to thank Prof Dr Ahmet Öztürk, Head of the Department of Biostatistics at the Faculty of Medicine, Erciyes University, and Funda İpekten, Research Assistant in the Department of Biostatistics and Medical Informatics at the Faculty of Medicine, Adiyaman University, for their contributions to the statistical evaluation of the study results.

## Author contributions

**Conceptualization:** Asli Gizem Çapar.

**Data curation:** Eda Nur Çetiner.

**Investigation:** Eda Nur Çetiner.

**Methodology:** Asli Gizem Çapar.

**Project administration:** Asli Gizem Çapar.

**Supervision:** Asli Gizem Çapar.

**Writing – original draft:** Asli Gizem Çapar, Eda Nur Çetiner.

**Writing – review & editing:** Asli Gizem Çapar.
